# 

**Published:** 1918-07-20

**Authors:** 


**July 20th, 1918.]** **[Price Twopence**

### The Hospital

The Workers' Newspaper of Administrative Medicine and Institutional Life, Administration, National Insurance and Health.

CONTENTS.

**The Medical Services of the Forces during the War** 335
**British Medical Students and Military Service** ... 342
**The Civil Hospitals of America** ... ... ... ... 341
"Gripped by Destructive Forces and Threatened with Disruption of their Organisations."
**London as a Post-Graduation Medical School** ... 336
**The Future of the Medical Profession...** ... ... 345
II. Teaching Hospitals. By Sir Bertrand Dawson, K.C.V.O., C.B., M.D.
**Temporary Artificial Legs (Illustrated)** ... ... 343
III. Amputation Below the Knee—Syme's Excluded.
**Hospital and Institutional News** ... ... ... 337
The Military Hospital Inspectors—A Great Step Forward at Blackburn—The Wider Issue—A Big Scheme at Cardiff—Feminists and the South London Hospital—The Day of Reckoning at the Guest Hospital—The Last Lap of Leeds Extension—Lord Durham's Charge Against the Red Cross—Scabies from Strap-hanging—Tommies and Town Planning—Excess Profits and Hospital Subscriptions—More Hospitals for Americans—Where Statistics Would be Useful—A Remarkable Inquiry at Bermondsey—Drugs Forbidden to Members of His Majesty's Forces—Small Hospitals and the Increased Duty on Alcohol—Should Unqualified Dispensing be Forbidden by Law?—This Week's Drug Market.
**Highlands and Islands Medical Service Board** ... 347
**The British Red Cross in France** ... ... ... 355
A Document of History.
**A Trumpet Call to Nurses** ... ... ... ... 348
The Gospel of Self-help by "Ierne."
**London Hospital Nurses...** ... .. ... ... 353
Justice for Probationers.
**The Matrons', Sisters', and Women's Departments** 349
Some Experiences of Training-school Life.
Round the Hospitals.
Queen Mary Accepts Half-a-million Gifts—The Nightingale School, Leeds—First College Centre Election—Fifteen Years as Cancer Sister—Soap and Alkali Waste in Laundries—Bracken for Pig-keepers.
**Editor's Letter Box** ... ... ... ... ... 354
A Certain Liveliness in Blackburn. Games for the Men.
**The Woman Workers' World** ... ... ... ... 354
**Parliament and the Profession** ... ... ... ... 356
**The Institutional Worker** ... ... ... ... I
The Steward's Department of a Large Hospital.

